# Brazilian Single-Center Experience with Aortic Root Replacement in 448 Patients: What Is the Best Technique?

**DOI:** 10.21470/1678-9741-2020-0043

**Published:** 2020

**Authors:** Fabrício José Dinato, Ricardo Ribeiro Dias, José Augusto Duncan, Fábio Fernandes, Felix José Alvares Ramirez, Charles Mady, Fabio B Jatene

**Affiliations:** 1 Department of Cardiovascular Surgery, Instituto do Coração (InCor), Hospital das Clínicas da Faculdade de Medicina da Universidade de São Paulo, São Paulo, SP, Brazil.; 2 Clinical Unit of Myocardiopathies and Aortic Diseases, Instituto do Coração (InCor), Hospital das Clínicas da Faculdade de Medicina da Universidade de São Paulo, São Paulo, SP, Brazil.

**Keywords:** Aorta, Aortic Diseases, Thoracic, Cardiac Surgical Procedures, Aortic Valve

## Abstract

**Introduction:**

The objective of this study was to evaluate whether a surgery with the use of valved conduit is capable of leading to better immediate and late results than those obtained by the valve-sparing aortic root reconstruction technique.

**Methods:**

Between January 2002 and June 2016, 448 patients underwent aortic root reconstruction. These were divided into three groups according to the technique used: 319 (71.2%) patients received mechanical valved conduits, 49 (10.9%) received biological valved conduits, and 80 (17.9%) underwent the valve-sparing aortic root reconstruction technique. The results were examined by univariate and multivariate analyses of Cox proportional hazards models with multiple logistic regression.

**Results:**

The hospital mortality rate was 7.5%. The mortality rates were 8.2%, 12%, and 2.5% in the mechanical valved conduit, biological valved conduit, and aortic valve-sparing groups, respectively, with no significant difference between groups (*P*=0.1). Thromboembolic complications and reoperation-free survival were also similar (*P*=0.169 and *P*=0.688). However, valve-sparing aortic root replacement was superior in terms of long-term survival (*P*<0.001), hemorrhagic-free survival (*P*<0.001), and endocarditis-free survival (*P*=0.048). Multivariate analysis showed that the following aspects had an impact on mortality: age > 70 years (*P*<0.001; hazard ratio [HR] 1.05), preoperative acute kidney injury (*P*<0.0042; HR 2.9), diagnosis of dissection (*P*<0.01; HR 2.0), previous cardiac surgery (*P*<0.027; HR 2.3), associated coronary artery bypass grafting (*P*<0.038; HR 1.8), reoperation for postoperative tamponade (*P*<0.004; HR 2.2) and postoperative acute kidney injury (*P*<0.02; HR 3.35).

**Conclusion:**

Valve-sparing technique seems to be the operation of choice, whenever possible, for aortic root reconstruction.

**Table:** 

Abbreviations, acronyms & symbols				
**AD**	**= Aortic dissection**		**COPD**	**= Chronic obstructive pulmonary disease**
**AMI**	**= Acute myocardial infarction**		**CPB**	**= Cardiopulmonary bypass**
**AR**	**= Aortic regurgitation**		**FC**	**= Functional class (New York Heart Association)**
**ARF**	**= Acute renal failure**		**HIV**	**= Human immunodeficiency virus**
**ARR**	**= Aortic root replacement**		**HR**	**= Hazard ratio**
**AS**	**= Aortic stenosis**		**MI**	**= Myocardial infarction**
**BMI**	**= Body mass index**		**MVS**	**= Mitral valve surgery**
**CABG**	**= Coronary artery bypass grafting**		**RF**	**= Renal failure**
**CI**	**= Confidence interval**		**SD**	**= Standard deviation**

## INTRODUCTION

Aortic root replacement (ARR) surgery using a valved conduit, whether mechanical or biological, is still the most commonly used technique for the correction of diseases affecting this aortic segment^[1]^. Although mechanical valves are traditionally used, these prosthesis expose patients to risks of complications related to thromboembolism and anticoagulation, in addition to infectious complications, which also occur with biological valved conduits.

Since the introduction of biological valved conduits by Griepp et al.^[2]^ in the 1990s, these conduits are available and avoid the adverse effects of prolonged anticoagulation. However, despite the longer durability of the new generation of bioprostheses available, there is a concern regarding the degeneration of the biological valve prostheses and the need for complex reoperation, which justifies the formal indication according to international guidelines in patients older than 70 years of age^[3]^.

Excellent results from valve-sparing ARR techniques have been widely published in recent years^[4]^. However, the complexity of the procedure and the need for adequate patient selection according to valvular pathology still limit this procedure for correction of aortic root diseases.

Through analysis of the results of different ARR techniques, the objective of this study was to evaluate whether operations involving the use of valved conduits lead to better immediate and late results than those obtained by the valve-sparing ARR technique.

## METHODS

From January 2002 to June 2016, 448 patients underwent ARR surgery. They were divided into three groups according to the ARR technique used: 319 (71.2%) patients underwent surgery using a mechanical valved conduit, 49 (10.94%) received a biological valved conduit, and 80 (17.86%) patients underwent ARR surgery with aortic valve preservation through the reimplantation technique.

Data were obtained through retrospective analysis of a prospectively constructed database from the Aorta Group of the Instituto do Coração - InCor, Hospital das Clínicas da Faculdade de Medicina da Universidade de São Paulo. The patients who were not followed up at the institution were evaluated through telephone contact. The study was approved by the institution's scientific and ethics committee and written consent from the patients was not required due to the characteristics of the study.

Three hundred seventy-two (83%) patients underwent aneurysm surgery, and 76 (17%) underwent chronic aortic dissection surgery. The mean patient age was 55 years, and 330 (73.66%) patients were men. The demographic characteristics of the patients under study are listed in Table 1. Patients with acute dissection were excluded from the study. Late follow-up was performed in 86% of the patients.

**Table 1:** Patients' characteristics.

Variables	Biological composite graft (n=49)	Mechanical composite graft (n=319)	Valve-sparing technique (n=80)	*P*-value
Mean age, years (mean ± SD)	68.5±12.0	54.2±14.2	51.4±16.6	< 0.001
Male, n (%)	36 (73.5)	236 (74.0)	58 (72.5)	0.964
BMI, kg/m^2^ (mean ± SD)	26.2±4.8	26.5±4.7	25.5 ± 4.6	0.375
Hypertension, n (%)	39 (76.0)	206 (64.6)	58 (72.5)	0.066
Diabetes mellitus, n (%)	8 (16.3)	28 (8.8)	7 (8.8)	0.238
Dyslipidemia, n (%)	17 (34.7)	70 (21.9)	17 (21.3)	0.130
Chronic RF, n (%)	14 (28.6)	29 (9.1)	7 (8.8)	< 0.001
Dialytic chronic RF, n (%)	0 (0.0)	1 (0.3)	1 (1.3)	0.493
Acute RF, n (%)	1 (2.0)	8 (2.5)	2 (2.5)	1
Smoking, n (%)	26 (53.0)	99 (31.0)	33 (41.3)	0.005
COPD, n (%)	11 (22.5)	26 (8.2)	5 (6.3)	0.003
Family history, n (%)	2 (4.1)	25 (7.8)	6 (7.5)	0.644
Dyspepsia, n (%)	5 (10.2)	24 (7.5)	8 (10.0)	0.673
Stroke with sequel, n (%)	0 (0.0)	4 (1.3)	1 (1.3)	1
Stroke without sequel, n (%)	2 (4.1)	12 (3.8)	0 (0.0)	0.185
HIV, n (%)	0 (0.0)	5 (1.6)	0 (0.0)	0.770
Cancer, n (%)	4 (8.2)	6 (1.9)	2 (2.5)	0.049
Coronary insufficiency, n (%)	17 (34.7)	51 (16.0)	15 (18.8)	0.007
Prior MI, n (%)	5 (10.2)	16 (5.0)	2 (2.5)	0.163
Reoperation, n (%)	6 (12.2)	86 (27.1)	2 (2.5)	< 0.001
Chest pain, n (%)	15 (30.6)	101 (31.7)	28 (35.0)	0.825
Prior atrial fibrillation, n (%)	8 (16.3)	49 (15.4)	3 (3.8)	0.021
Marfan syndrome, n (%)	2 (4.1)	21 (6.6)	13 (16.3)	0.010
Bicuspid aortic valve, n (%)	13 (26.5)	59 (18.5)	8 (10.0)	0.051
Ejection fraction, (mean ± SD)	0.55±0.12	0.58±0.12	0.59±0.09	0.215
Aortic diameter, mm (mean ± SD)	54.9±7.3	58.5±11.2	54.4±8.9	0.002
**Aortic regurgitation, n (%)**				0.301
Moderate/severe AR, n (%)	39 (81.3)	232 (73.4)	55 (18.8)	
Aortic stenosis, n (%)				0.013
Moderate/severe AS, n (%)	3 (6.3)	12 (3.7)	0 (0.0)	
**Cardiac insufficiency, n (%)**				
FC III/IV, n (%)	18 (36.7)	83 (26.1)	7 (8.8)	< 0.001
**Indication for surgery, n (%)**				0.347
Aneurysm, n (%)	45 (93.9)	251 (79.0)	76 (95.0)	< 0.001
Type A chronic AD, n (%)	3 (6.1)	68 (21.3)	5 (6.3)	< 0.001

AD=aortic dissection; AR=aortic regurgitation; AS=aortic stenosis; BMI=body mass index; COPD=chronic obstructive pulmonary disease; FC=functional class (New York Heart Association); HIV=human immunodeficiency virus; MI=myocardial infarction; RF=renal failure; SD=standard deviation

The indications for surgical treatment of aortic root diseases were in accordance with the last American and European guidelines of 2010 and 2014[5,6], respectively. The operative technique used for ARR with either a mechanical or a biological valve conduit was performed using the modified Bentall procedure. The aorta was transected at the sinotubular junction and the coronary ostia excised with a cuff of aortic wall. The aortic root and valve were excised and then replaced by suture of a valvar conduit onto the annulus with the use of interrupted pledgeted polyester 2-0 suture. The coronary ostia were reimplanted and the distal aorta was anastomosed.

A self-made biological composite graft was chosen for those older than 60 years of age and those in whom anticoagulation was contraindicated. After measuring the aortic annulus size and selecting the appropriate-size valve prosthesis, the biological valve was sutured intraoperatively with the vascular conduit using three single polypropylene sutures.

The reimplantation technique (David III procedure)[7] was performed in patients without significant valvular regurgitation, those with valvular regurgitation secondary to annular dilatation, and those with valve regurgitation due to prolapse of one or more leaflets susceptible to plasty. The valve-sparing procedure was performed with the dissection of the aortic root as low as possible. Commissural traction stitches were applied, and the left and right coronary buttons were prepared. The aortic sinuses were resected preserving a 3-5 mm rim of aortic wall just above the annulus alongside each of the three commissures. Three subcommissural 2-0 polyester sutures from inside to outside were placed below the aortic annulus and more three 2-0 polyester stitches were placed from inside to outside just below the nadir leaflet insertion. These sutures were passed through the base of the vascular prosthesis respecting their proximal positioning. Then a 4-0 polypropylene running suture for valve reimplantation was performed passing the suture from outside the prosthesis to inside and through the aortic wall, staying close to the annulus, and then back out of the prosthesis. Coronary reimplantation was performed with a 5-0 polypropylene running suture and darts were placed between each commissure to create bulges in the graft. The intraoperative characteristics are listed in Table 2.

**Table 2:** Intraoperative data.

Variables	Biological composite graft (n=49)	Mechanical composite graft (n=319)	Valve-sparing technique (n=80)	*P*-value
CPB time, min (mean ± SD)	125.7±26.5	134.0±35.0	166.5±28.4	< 0.001
Myocardial ischaemic time, min (mean ± SD)	103.4±22.3	108.0±27.4	144.7±23.3	< 0.001
Associated procedures, n (%)				
CABG, n (%)	11 (22.5)	39 (12.2)	7 (8.8)	0.068
MVS, n (%)	7 (14.3)	18 (5.6)	6 (7.5)	0.083
Stent grafting via aortic arch, n (%)	1 (2.0)	19 (6.0)	3 (3.8)	0.614

CABG=coronary artery bypass grafting; CPB=cardiopulmonary bypass; MVS=mitral valve surgery; SD=standard deviation

The results analyzed were hospital complications and mortality, endocarditis-free survival, hemorrhagic and thromboembolic complication-free survival, reoperation-free survival, and late survival.

The follow-up time ranged from one month to 11 years, with an average of 2.63 years. When we analyzed the groups separately, the mean follow-up time of the groups of patients who underwent ARR with valve-sparing, mechanical, and biological valved conduits was 3.5, 2.5, and 1.6 years, respectively.

### Statistical Analysis

For the data analysis, continuous variables are expressed as the mean ± standard deviation and categorical variables are described as percentages. Continuous variables were analyzed using the nonparametric Kruskal-Wallis test. The chi-square test and Fisher's exact test, when appropriate, were used to analyze the categorical variables. The univariate and multivariate analyses of Cox proportional hazards models were used to study the associations between risk factors and survival, and multiple logistic regression was performed through the stepwise selection process. Survival curves and event-free survival curves were estimated using the Kaplan-Meier method and the log-rank test. *P*<0.05 was considered statistically significant.

## RESULTS

The overall hospital mortality rate was 7.5%. The hospital mortality rate was 8.2% (26/319) in the group of patients who underwent ARR with a mechanical valve conduit, 12% (6/49) in the group of patients who underwent ARR with a biological valve conduit, and 2.5% (2/80) in the valve-sparing ARR group, with no significant difference between the groups (*P*=0.1).

Hospital deaths were due to low cardiac output in 10 (2.2%) patients, hemorrhagic shock in eight (1.78%) patients, pneumonia in five (1.1%) patients, low cardiac output associated with sepsis in four (0.9%) patients, septic shock in three (0.66%) patients, mediastinitis in two (0.45%) patients, mesenteric ischemia in one (0.22%) patient, and one (0.22%) patient died for unknown reasons.

In terms of hospital complications, low cardiac output was the only hospital complication that was different between the groups (*P*=0.014). Cardiac output was significantly better in the group in which the valve was preserved, despite the longer cardiopulmonary bypass duration and myocardial ischemia time required to perform the procedure (Table 3).

**Table 3:** Postoperative complications.

Variables	Biological composite graft (n=49)	Mechanical composite graft (n=319)	Valve-sparing technique (n=80)	*P*-value
Re-exploration for bleeding, n (%)	9 (11.4)	6 (15.4)	3 (7.5)	0.311
Low cardiac output, n (%)	8 (10.1)	8 (20.5)	0 (0.0)	0.002
Wound infection, n (%)	8 (10.1)	3 (7.7)	5 (12.5)	0.712
Mediastinitis, n (%)	1 (1.3)	0 (0.0)	1 (2.5)	1
Pneumonia, n (%)	22 (27.9)	7 (18.0)	15 (37.5)	0.053
Urinary tract infection, n (%)	10 (12.7)	4 (10.3)	6 (15.0)	0.737
Sepsis, n (%)	19 (24.1)	10 (25.6)	9 (22.5)	0.744
Prolonged mechanical ventilation, n (%)	7 (8.9)	4 (10.3)	3 (7.5)	0.712
ARF without dialysis, n (%)	31 (39.2)	10 (25.6)	21 (52.5)	0.015
ARF with dialysis, n (%)	6 (7.6)	3 (7.7)	3 (7.5)	1
Delirium, n (%)	3 (3.8)	1 (2.6)	2 (5.0)	1
Stroke (permanent deficit), n (%)	4 (5.1)	1 (2.6)	3 (7.5)	0.615
Spinal cord injury, n (%)	2 (2.5)	2 (5.1)	0 (0.0)	0.241
AMI, n (%)	2 (2.5)	1 (2.6)	1 (2.5)	1
Atrial arrhythmias, n (%)	17 (21.5)	8 (20.5)	9 (22.5)	0.830
In-hospital mortality, n (%)	16 (20.3)	12 (30.8)	4 (10.0)	0.022

AMI=acute myocardial infarction; ARF=acute renal failure

**Fig. 1:**
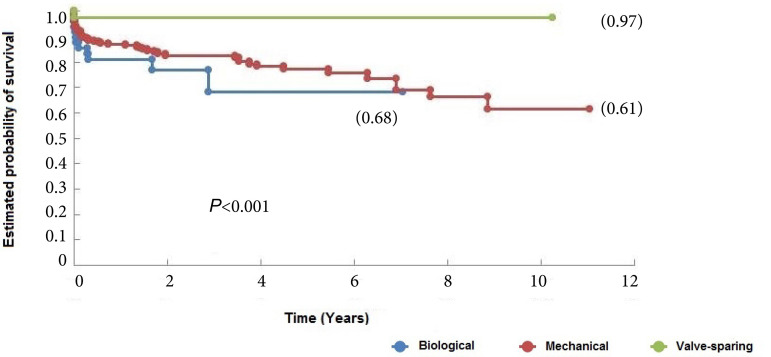
Survival curves of patients undergoing aortic root replacement surgery using mechanical and biological valved conduits and with valve-sparing techniques.

When late survival was evaluated, the group of patients who underwent valve-sparing ARR had longer survival (97%) during the follow-up period (*P*<0.001) (Figure 1). The median-term and the one-year survival rates of the entire cohort was 68.8% (58.2% - 79.4% 95% confidence interval [CI]) and 88,3% (85,1% - 91,5% 95% CI), respectively (*P*<0,001) (Table 4).

**Table 4:** Survival rates.

Techniques	Median-term survival rate (95% CI)	One-year survival rate (95% CI)	*P*<0.001
Entire cohort	68.8% (58.2 - 79.4%)	88.3% (85.1 - 91.5%)	
Biological composite graft	68.3% (48.3 - 88.3%)	80.9% (69.3 - 92.5%)	
Mechanical composite graft	61.4% (47.6 - 75.2%)	87% (83 - 91%)	
Valve-sparing technique	97.5% (93.9 - 100%)	97.5% (93.9 - 100%)	

CI=confidence interval

**Fig. 2:**
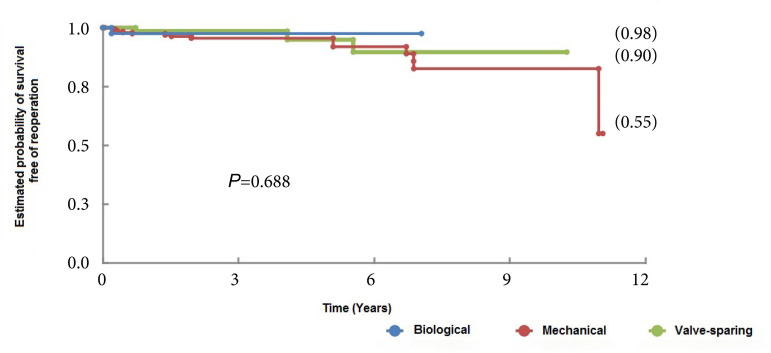
Reoperation-free survival curves of patients undergoing aortic root replacement surgery using mechanical and biological valved conduits and with valve-sparing techniques.

Regarding the reoperation-free survival, similar behavior was observed between the groups, demonstrating that aortic valve preservation was not associated with a greater need for reoperation during the follow-up period (*P*=0.688) (Figure 2).

**Fig. 3:**
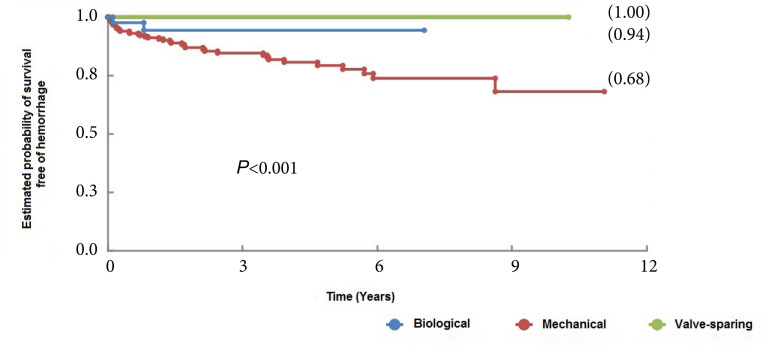
Hemorrhagic complication-free survival curves of patients who underwent aortic root replacement surgery using mechanical and biological valved conduits and with valve-sparing techniques.

Concerning hemorrhagic complications, there was a difference between the groups. The rate of hemorrhagic complications was significantly higher in the group of patients treated with a mechanical valve conduit (*P*<0.001). These complications occurred in 10% of the patients (32/319), and in 1.9% of the patients (6/319) they were the cause of death (two patients died of hemorrhagic stroke, two patients died of upper gastrointestinal bleeding, one patient died of cardiac tamponade, and one patient died of a spinal cord hematoma) (Figure 3).

**Fig. 4:**
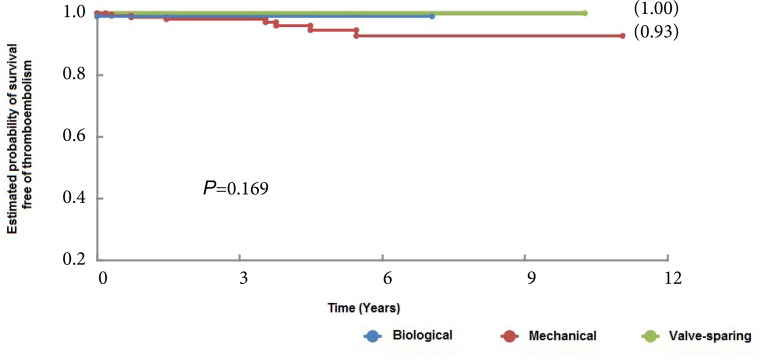
Thromboembolic complication-free survival curves of patients who underwent aortic root replacement surgery using mechanical and biological valved conduits and with valve-sparing techniques.

There was no difference in thromboembolic complications during the follow-up period, as shown in Figure 4 (*P*=0.169).

**Fig. 5:**
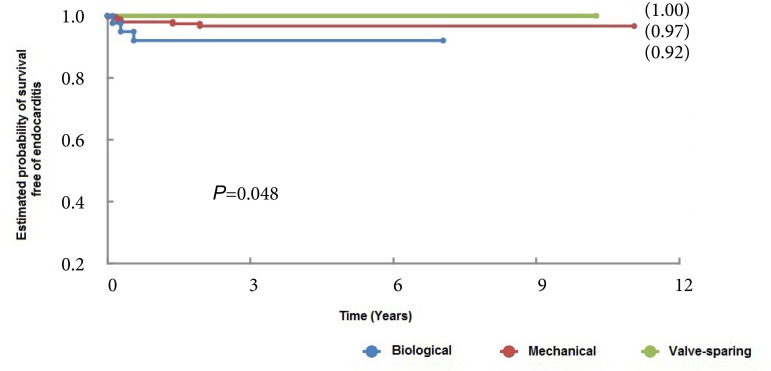
Endocarditis-free survival curves of patients undergoing aortic root replacement surgery using mechanical and biological valves and with valve-sparing techniques.

The occurrence of late endocarditis was lower in the group in which the aortic valve was preserved (*P*=0.048). Of the 10 patients affected (all of whom underwent ARR with a valved conduit), 60% required surgery (Figure 5).

The multivariate analysis showed that the following factors had an impact on mortality: age > 70 years, with a hazard ratio (HR) of 1.05 (*P*<0.0001); preoperative acute kidney injury, with a HR of 2.9 (*P*<0.0042); diagnosis of dissection, with a HR of 2.0 (*P*<0.01); history of previous cardiac surgery, with a HR of 2.3 (*P*<0.027); need for associated coronary artery bypass grafting, with a HR of 1,8 (*P*<0.038); need for reoperation for postoperative tamponade, with a HR of 2.2 (*P*<0.004); and acute kidney injury during the postoperative period, with a HR of 3.35 (*P*<0.02).

## DISCUSSION

ARR using valved conduits for treatment of aortic root diseases presents a low mortality rate and good long-term results. The overall operative mortality (death within 30 days of surgery) of ARR with valved conduits in recent publications varies from 0.7% to 11%, with survival rates ranging from 76% to 91.8% in five years and 62% to 76% in 10 years^[8]^. However, in recent years, several series have shown that valve-sparing ARR is an interesting alternative for patients with normal or slightly altered aortic valve leaflets, with a reoperation-free survival rate of 94% to 97% of patients^[8]^.

David et al.^[9]^ published a study of 151 patients who underwent valve-sparing ARR in patients with aneurysms of the aortic root and reported 99.1% aortic valve reoperation-free survival when the reimplantation technique was used and 83% late survival for patients with root aneurysms at eight years of follow-up. Dias et al.^[10]^ presented the results of 78 patients who underwent valve-sparing ARR during a follow-up period of 1621±1156 days. When comparing patients who underwent aortic valve repair with those who did not undergo this procedure, they observed that aortic valve repair did not increase the risk of reoperation of the valve and was a very safe and long-lasting approach.

In this study, we presented the results of our experience in ARR and despite the difference between the groups, we compared the ARR techniques with valved conduits (mechanical or biological) and the valve-sparing reimplantation technique. Our data indicated 96,5% freedom from reoperation in valve-sparing group during follow-up, with only two patients indicated for aortic valve approach, demonstrating that aortic valve preservation was not associated with a greater need for reoperation. Actually, in this study, we observed that valve sparing aortic root reimplantation, when feasible, is an excellent alternative to composite conduits with mechanical or bioprostheses, with favorable short and medium-term outcomes.

Although the classic technique uses mechanical valved conduits, tubes constructed with biological prostheses have also presented good results. Etz et al.^[11]^ published a series of 275 patients, with a mean age of 69 years, who underwent ARR surgery with biological valved conduits and found a hospital mortality of 6.2% and a need for reoperation due to structural deterioration of the prosthesis in only one patient (0.4%) 12 years after surgery. Lehr et al.^[12]^ reported a study of 144 patients who underwent ARR surgery and compared the results between the use of mechanical and biological valved conduits. They described an operative mortality of 2.1%; one- and five-year survival rates for the mechanical group were 96.0% and 89.0%, respectively, *vs*. 93.0% and 84.0% for the biological group, concluding that mortality rate and complications related to the type of prosthesis used were similar in both groups at five years of follow-up.

In our experience, we found a significantly lower rate of hemorrhagic complications in the group of patients treated with biological valved conduit, with similar rate of endocarditis between mechanical and biological valve prosthesis. In addition, at the present study, we demonstrated a similar hospital mortality and overall probability of survival for patients with mechanical and biological composite grafts, although without an age-matched population analysis. Therefore, ARR with a biological valve seems to be an excellent alternative for those with contraindications for anticoagulation or older patients, since they are associated with low probabilities of structural prosthesis deterioration and need for reoperation, in addition to low rates of hemorrhagic or thromboembolic complications compared with mechanical valves. Besides, biological valved conduits have become an attractive option to consider in cases of posterior transcatheter valve-in-valve implantation, which is increasingly considered in clinical practice.

Gaudino et al.^[13]^ published a series of 890 patients who underwent ARR with a mechanical composite valved graft, biological composite valved graft, or a valve-sparing reconstruction. Their operative mortality was 0.2%, five-year survival was 89.4% and, in the propensity-matched groups, the type of operation performed did not affect in-hospital and late outcomes.

In our study, during the follow-up period, valve-sparing ARR was superior to the other techniques in terms of long-term survival, freedom from hemorrhagic complications, and freedom from endocarditis, and there was no difference between ARR techniques regarding the need for reoperation.

Karck et al.^[14]^, in a series of 119 patients with Marfan syndrome, compared composite graft replacement with mechanical valve conduits and aortic valve-sparing reimplantation and demonstrated freedom from reoperation and death after one year of 97% and 97% for ARR with mechanical valved conduit and 95% and 100% for valve-sparing by reimplantation technique, with no significant difference between groups. Although similar survival contrasts with our results, they also showed lower risk of bleeding complications in the valve-sparing group and similar freedom from reoperation.

Svensson et al.^[15]^ described a large single-institution series of 957 patients who underwent four aortic root procedures and showed less risk of valve-related complications, such as bleeding and endocarditis, and lowest late risk of reoperation in the valve-sparing group in a follow-up of 5.3 years. They also described a worst survival rate in patients who underwent biological composite graft ARR, probably due to differences in patients’ characteristics.

Similarly, it must be noted that although we found a superior long term-survival in the valve-sparing group, any difference in survival can be attributed to differences in patient demographic characteristics, particularly age, prevalence of comorbidities and aortic pathology.

Most risk factors for mortality in our multivariate analysis were related with age, preoperative and postoperative acute kidney injury, prior cardiac surgery, a diagnosis of dissection, coronary artery bypass grafting associated with aortic surgery, and postoperative tamponade, and we strongly believe that mortality rate is influenced by an amount of co-morbidities rather than the surgical technique.

Furthermore, despite the superior results observed for valve-sparing ARR technique during the follow-up period, mainly for younger patients with aortic root aneurysm and aortic valve regurgitation with normal leaflets or susceptible plasty, it must be mentioned that, for those surgeons who are not familiar with valve-sparing operation, ARR with composite conduits is certainly still a valuable option. In addition, the valve-sparing ARR technique is not feasible for all patients because of compromised leaflets. However, whenever possible, the best late survival achieved with valve preservation needs to be considered, and surgeons should be prepared to perform this procedure.

### Limitations

We must highlight some limitations of this study. Mainly, because it was a retrospective study in which randomization for the aortic root reconstruction technique applied was not possible. In addition, the study had a short postoperative follow-up time and the groups were not completely similar. The short follow-up time was due to the fact that a greater number of patients was operated few years before and loss of follow-up occurred regarding patients undergoing surgery at the beginning of the study period.

## CONCLUSION

Due to the limitations mentioned above, it was not possible to definitively clarify which technique is the best for ARR. However, according to our results, we can conclude that the valve-sparing technique seems to be the operation of choice, whenever possible, for ARR.

**Table:** 

Authors' roles & responsibilities	
FJD	Substantial contributions to the conception or design of the work; acquisition, analysis, and interpretation of data for the work; drafting the work and revising it critically for important intellectual content; final approval of the version to be published
RRD	Substantial contributions to the conception or design of the work; acquisition, analysis, and interpretation of data for the work; drafting the work and revising it critically for important intellectual content; final approval of the version to be published
JAD	Substantial contributions to the conception or design of the work; acquisition, analysis, and interpretation of data for the work; drafting the work and revising it critically for important intellectual content; final approval of the version to be published
FF	Agreement to be accountable for all aspects of the work in ensuring that issues related to the accuracy or integrity of any part of the work are appropriately investigated and resolved; final approval of the version to be published
FJAR	Agreement to be accountable for all aspects of the work in ensuring that issues related to the accuracy or integrity of any part of the work are appropriately investigated and resolved; final approval of the version to be published
CM	Agreement to be accountable for all aspects of the work in ensuring that issues related to the accuracy or integrity of any part of the work are appropriately investigated and resolved; final approval of the version to be published
FBJ	Agreement to be accountable for all aspects of the work in ensuring that issues related to the accuracy or integrity of any part of the work are appropriately investigated and resolved; final approval of the version to be published
